# Protective effect of melatonin on cadmium-induced changes in some maturation and reproductive parameters of female Prussian carp (*Carassius gibelio* B.)

**DOI:** 10.1007/s11356-018-1308-8

**Published:** 2018-01-26

**Authors:** Ewa Drąg-Kozak, Magdalena Socha, Grzegorz Gosiewski, Ewa Łuszczek-Trojnar, Jarosław Chyb, Włodzimierz Popek

**Affiliations:** Department of Ichthyobiology and Fisheries, University of Agriculture in Krakow, ul. Spiczakowa 6, 30-199 Krakow-Mydlniki, Poland

**Keywords:** Melatonin, Cadmium, LH, Bioaccumulation, Depuration, Prussian carp

## Abstract

The aim of this study was to determine whether melatonin (Mel), which is a known antioxidant and free radical scavenger, could perform the role of a preventive agent against the toxic effects of cadmium (Cd^2+^) on mortality, fish growth, gonadosomatic index (GSI), luteinizing hormone (LH) secretion, the response to hormonal stimulation of spawning, and also tissue accumulation of Cd in Prussian carp females. These females received melatonin implants and were exposed to 0.4 or 4.0 mg/L of Cd (as CdCl_2_·2.5H_2_O) over either a 5- or 3-month period, followed by further 2 months of purification in clear water. Negative changes caused by exposure to cadmium in the water were as follows: higher fish mortality, lower body weight, increased accumulation of cadmium in the brain and ovary, lowered GSI, impaired spontaneous LH secretion during exposure, and impaired LH secretion during stimulation of spawning. All of these effects were observed in the group of fish exposed to 0.4 and/or 4.0 mg Cd/L but did not occur or were less pronounced in the groups exposed to cadmium in the presence of melatonin released from the implants. During depuration, in the group of fish which had been exposed to the highest Cd concentration, we observed a significant improvement in fish survival rate, body growth, inhibition of further cadmium accumulation in tissues, and gradual return of spontaneous LH secretion as well as normalization of the GSI value to the control group levels. In conclusion, these findings indicate that melatonin can be a preventive agent for some toxic effects on fish reproduction induced by environmental cadmium contamination.

**Electronic supplementary material:**

The online version of this article (10.1007/s11356-018-1308-8) contains supplementary material, which is available to authorized users.

## Introduction

Cadmium (Cd^2+^) is one of the most important toxic heavy metals and a non-essential element widely used in industrial and agricultural practices such as fertilizers and pesticides (Satarug et al. 2003). Cadmium concentrations in environmental water may be considerable and range from 0.01 to over 16.1 mg/L (Peng et al. 2009). Cadmium is known as a non-biodegradable metal and its rapid accumulation in organisms, but very slow elimination is an ecologically significant problem. One of the main reasons for the increased presence of cadmium in organisms is the ability to accumulate cadmium by induction of the metal binding protein, metallothionein (MT), which is believed to influence the uptake, distribution, and toxicity of cadmium (Asagba et al. 2008). Furthermore, Cd bound to MT is responsible for Cd accumulation in tissues and the long biological half-life in the body. The biological half-life of this metal may be as long as 10 to 30 years (Romero et al. 2011; Sharma et al. 2014).

Toxic chemicals released into surface water may influence the growth and reproduction of aquatic organisms, especially fish (Szczerbik et al. 2006; Okorie et al. 2014). Numerous studies carried out on fish proved the toxic effect of cadmium on the nervous, endocrinal, and reproductive systems. This metal can penetrate the blood-brain barrier and accumulate in brain tissues (Pillai et al. 2003). The hypothalamo-pituitary-gonadal axis seems to be the main target of toxic cadmium influence. This metal may act at various sites on this axis altering the reproductive endocrine function (Tilton et al. 2003). The hypothalamus produces gonadotropin releasing hormone (GnRH), which stimulates the pituitary, to produce and release gonadotropin hormones such as follicle-stimulating hormone (FSH) and luteinizing hormone (LH), which regulates gonadal functions such as the induction of final oocytes maturation and ovulation (Yaron and Levavi-Sivan 2011). It has been shown that cadmium in fish affects LH secretion, also causing hypertrophy, vacuolization, and degeneration of gonadotropic cells (Szczerbik et al. 2006). Numerous experiments have proved that cadmium has negative effects on ovarian functions in fish. This metal causes the ovary structure to disintegrate, oolemma necrosis, and follicular cells hypertrophy. In the early gonadal recrudescence phase, cadmium increased steroid ovarian hormone secretion and the premature growth of the ovary (El-Ebiary et al. 2013).

Melatonin, a hormone principally released by the pineal organ, functions as a non-enzymatic antioxidant and lowers oxidative stress by free radical scavenging or suppression of their synthesis by regulation of antioxidative enzyme activity (Eybl et al. 2006; Romero et al. 2011). Melatonin is also synthesized in other cells, tissues, and organs, such as the retina, lens, pituitary gland, bone marrow cells, gut, and skin, expressing their paracrine and autocrine activities (Acuña-Castroviejo et al. 2014). In addition, locally produced melatonin does directly protect cells against free radicals (Manchesterem et al. 2015). In fish, melatonin has also been related to smoltification, reproduction, locomotor activity, sedation, skin pigmentation, oxygen consumption, osmoregulation, thermoregulation, food intake, and shoaling behavior (Kulczykowska et al. 2006; Falcón et al. 2007).

To date, a great deal of evidence has shown the role of melatonin in fish reproduction, by acting at different levels of the reproductive axis. Melatonin receptors have been found to be localized in the hypothalamus and pituitary gland, as well as in the ovaries of fish (Falcón et al. 2007; Chattoraj et al. 2009). Its influence, depending on species-specific and numerous external factors as season, lighting conditions, or water temperature, may either suppress or stimulate gonad maturation (Falcón et al. 2007).

In studies conducted on mammals, it was shown that melatonin may effectively modulate the neurotoxic action of cadmium on the hypothalamic-pituitary-gonadal axis (Poliandri et al. 2006; Alonso-González et al. 2007; Romero et al. 2011; Xu et al. 2016). One of the mechanisms of melatonin action is its ability to intercept and bind cadmium, thus preventing its toxic influence on the hypothalamus and pituitary gland (Limson et al. 1998, Cano et al. 2007).

No data exist concerning the influence of melatonin on the accumulation and toxicity of cadmium as well as its impact on the hypothalamic-pituitary-gonadal axis in fish exposed to this metal. Considering the literature data on cadmium and melatonin interactions in mammals, it seems reasonable to carry out studies aiming to explain whether melatonin, a non-enzymatic compound with anti-oxidative properties, also protects fish organisms against cadmium toxicity. Therefore, the main purpose of the present work was to investigate the possible modulatory role of melatonin against cadmium-induced neurotoxic effects on spontaneous LH secretion and LH secretion hormonally stimulated with an analogue of salmon GnRH (sGnRH-a). An additional objective was to determine the effect of melatonin on the accumulation and elimination of Cd in the brain and ovary during 5 months’ exposure to this metal or 3 months’ exposure and a 2-month depuration period.

## Material and methods

### Experimental fish

The research was conducted at the Fishery Experimental Station in Mydlniki at the Department of Ichthyobiology and Fisheries of the University of Agriculture in Cracow, Poland. Three hundred and forty-three Prussian carp (*Carassius gibelio* B.) females, originating from the Mydlniki Station, were used in the study. The Prussian carp was chosen as an experimental model due to that most European populations are monosex populations, living as triploid females that reproduce gynogenetically (Boroń et al. 2011).

The fish were kept in 7 tanks (700 L, 49 fish per tank) under a simulated, natural photoperiod (L/D = 14:10). The physicochemical characteristics of the experimental water, from the Municipal Waterworks in Cracow, were as follows: temperature 18 °C, pH = 7.6, dissolved oxygen concentration 9.5 mg/L, and water hardness 186 mg CaCO_3_/L. In addition, the water was filtered and aerated. During the 3-month acclimation period and throughout the experiment, the fish were hand-fed with commercial dry pellets. These pellets of complete feed for Prussian carp were produced by Aller Aqua using grains, fish meal, oilseed, vitamin, and mineral supplements. The feed was composed of 12% crude fat, 37% crude protein, and 32.5% carbohydrate. Throughout the study, the fish were fed once daily a ration that amounted to 3% of their body weight. At the age of 3 years, the fish (mean body weight of 204.65 **±** 12.58 g and body length of 23.25 **±** 0.49 cm) were divided into seven groups: control group—nominally zero cadmium, group Mel—the fish received intramuscular melatonin implants, blank group—the fish were sham-injected, group 0.4 mg Cd/L + Mel—the fish received intramuscular melatonin implants and were exposed to cadmium in water, group 0.4 mg Cd/L—the fish were exposed to cadmium in water, group 4.0 mg Cd/L+Mel—the fish received intramuscular melatonin implants and were exposed to cadmium in water, and group 4.0 mg Cd/L—the fish were exposed to cadmium in water. The stock solution of Cd was prepared by dissolving analytical grade of cadmium chloride (CdCl_2_·2.5H_2_O) in distilled water. The concentration of cadmium was expressed in terms of Cd ion in milligrams per liter. The water in the tanks was changed every 2 days to maintain a constant metal concentration. The water cadmium levels to which Prussian carp were exposed in the present experiment were chosen based on the reported concentrations of this metal in environmental water samples, which range from 1 to over 16.1 mg/L (Tilton et al. 2003; Peng et al. 2009). Analysis of cadmium concentrations in water samples showed the following mean levels: control group, Mel and blank—cadmium concentrations were below detection limits, group 0.4 mg Cd/L + Mel—0.39 (± 0.03) mg/L, group 0.4 mg Cd/L—0.41 (± 0.03) mg/L, group 4.0 mg Cd/L + Mel 4—3.98 (± 0.38) mg/L, and group 4.0 mg Cd/L—4.01 (± 0.39) mg/L.

An implanter was used to administer the implants intramuscularly 1 cm below the dorsal fin on the right side of the body. A description of the surgical procedure used to implant melatonin in the fish is given by Porter et al. (1998) and Mazurais et al. (2000). Melatonin implants containing 18 mg of melatonin (Ceva Santa Animale France) were used to elevate plasma melatonin levels in fish. A lot of research studies indicate that circulating melatonin levels were measured after the implantation in fish show the increase from 4.0 pg/mL (control) to 1 ng/mL (melatonin implanted) during 5 months (Aarseth et al. 2010; Sönmez et al. 2014).

The fish were exposed to cadmium for a period of 3 or 5 months. The groups (control, Mel, blank—not treated with Cd) were kept under the same conditions for 5 months. In the case of experimental groups treated with Cd, each of them was divided into two groups of fish after 3 months of exposure. One of them was kept under the same treatment regime, while the second one (groups: 0.4 mg Cd/L + Mel-dep, 0.4 mg Cd/L-dep, 4.0 mg Cd/L + Mel-dep, and 4.0 mg Cd/L-dep) was transferred to clean water and experienced a depuration period which lasted for the next 2 months until the end of the experiment (Table 1S).

### Locomotor activity and growth

The behavioral changes in the fish were evaluated for anomalies such as difficulty in breathing and gathering around the ventilation filter, slowness in motion, and sinking down to the bottom. Fish from each aquarium were individually weighed before exposure and after 1, 2, 3, 4 and 5 months of the experiment.

### Cd determinations

Before the exposure and at 1, 2, 3, 4, and 5 months of the experiment, the brain and ovary samples were collected from seven randomly harvested fish from each group. These tissues were subjected to preliminary mineralization in the presence of 10 mL, a 3:1 *v*/*v* mixture of nitric acid (65% HNO_3_) and perchloric acid (70% HClO_4_) for about 20 h. The samples were then heated with a Velp 20/26 digester by gradually increasing the temperature up to 180 °C for 6–7 h. The clear liquid obtained was then diluted with deionized water to 10 mL and then assayed for the concentration of cadmium using a Unicam 929 atomic absorption spectrometer (Agemian et al. 1980). These concentrations were read from the standard curve generated, using the standards based on atomic absorption standards made at the Office of Weights and Measures in Warsaw. The results were presented in milligrams of Cd per kilogram of wet tissue weight (ww).

### LH analysis

In order to investigate the influence of cadmium and/or melatonin on spontaneous LH secretion, the concentration of this hormone in blood plasma was determined after each month of the experiment. During the natural environment period of spawning (which occurred after 3 months of exposure), analysis of LH secretion following hormonal stimulation of the fish was performed. Seven days before the planned experiment, seven fish from each group were placed in 300 L aquariums (the division into groups was maintained) at a water temperature of 20 °C. After blood collection (for basal level), each fish was intraperitoneally injected with a salmon gonadotropin releasing hormone analogue (sGnRHa, 10 μg sGnRHa/0.5 mL/kg b.wt; Bachem Feinchemikalien AG, Switzerland) and blocker of D-2 dopamine receptors—pimozide (5 μg/0.5 mL/kg b.wt; Sigma-Aldrich, Germany) to stimulate LH secretion from the pituitary gland. To evaluate the effect of cadmium and/or melatonin on that stimulation, blood samples were collected from fish to determine the LH level in blood plasma. Blood samples (about 100 μL from each fish) were collected at the beginning of the experiment (basal level) and at 6, 12, and 24 h after the injection from the caudal vein with a 1-mL heparinized syringe. The blood samples were centrifuged at 13,000×*g* for 3 min in Eppendorf tubes, and blood plasma samples were kept at − 20 °C until the luteinizing hormone (LH) was determined using the ELISA method (Kah et al. 1989). The specific antibody and standard LH hormone were donated by Dr. Bernard Breton (INRA, France). The fish were anesthetized with Propiscin (0.3 mL/L; IRS, Żabieniec, Poland) prior to all the manipulations.

### GSI analysis

Before the exposure and at 1, 2, 3, 4, and 5 months of the experiment, the value of GSI was calculated during collection from the brain and ovary for analysis of cadmium concentration, using the formula: gonad weight (g)/whole body weight (g) × 100 (Zeyl et al. 2014).

### Statistical analysis

The results of the analysis were expressed as the mean ± standard error of the mean (SEM). The results were analyzed using one-way ANOVA, and the Mann-Whitney procedure was used to determine significant differences between the means for the control and experimental groups, as well as between groups at successive months of exposure. The relationship between the cadmium concentration in the brain and ovary and the dose of exposure was calculated using Spearman’s correlation coefficients. To observe the relationships between GSI and Cd concentration in gonad tissue, Spearman’s correlation coefficients were calculated. Moreover, the relationships between LH secretion and Cd doses during the exposure were calculated using Spearman’s correlation coefficients. The differences between the means were determined as significant at *p* ≤ 0.05.

## Results

### Mortality, body weight, and behavior

The highest mortality of fish (3%) was found in the group exposed to the higher cadmium dose compared to other groups. Melatonin-treated fish were found to be healthy throughout the observation period, and there was no mortality noted.

At the beginning of the experiment, the differences in average fish body weight between the groups were not statistically significant. Successive measurements of fish body weight showed gradual inhibition of their growth in the group exposed to the highest cadmium dose in water (4.0 mg Cd/L). After 3 and 4 months of exposure, the lowest body weight was observed in the group exposed to the highest cadmium dose in comparison with other months. At the end of the experiment (after 5 months’ exposure), the average body weight of fish in the group exposed to 4.0 mg Cd/L was significantly lower in comparison to the 0.4 mg Cd/L, Mel, and blank groups (Table 1). Melatonin did not significantly affect the body weight of the fish.

**Table 1:** Comparison of body weight (g) and LH spontaneous secretion (ng/mL) in Prussian carp females (mean ± SD) during 5 months of water exposure to cadmium and 2 months of depuration

Month	Control	Mel	Blank	0.4 mg Cd/L + Mel	0.4 mg Cd/L	4.0 mg Cd/L + Mel	4.0 mg Cd/L	0.4 mg Cd/L + Mel-dep	0.4 mg Cd/L-dep	4.0 mg Cd/L + Mel-dep	4.0 mg Cd/L-dep
Body weight (g)											
0	218.0 ± 14.76 Aa	205.1 ± 12.73 Aa	198.0 ± 8.26 Aab	226.6 ± 16.03 Aa	196.6 ± 7.79 Aa	210.9 ± 13.88 Aa	218.0 ± 14.63 Aa	NT	NT	NT	NT
1	219.1 ± 14.64 ABa	206.9 ± 10.25 Aab	210.3 ± 13.10 Aab	210.3 ± 11.17 Aa	212.3 ± 18.14 Aa	174.6 ± 7.05 Ba	213.0 ± 1.27 Aa	NT	NT	NT	NT
2	228.6 ± 17.87 Aa	218.6 ± 13.51 Aab	201.1 ± 9.90 ABa	217.7 ± 15.12 Aa	167.7 ± 10.47 Ba	186.0 ± 11.74 Aa	185.1 ± 15.22 ABab	NT	NT	NT	NT
3	205.4 ± 10.00 ABa	210.6 ± 15.35 ABab	209.4 ± 10.21 ABab	211.1 ± 6.73 Aa	184.0 ± 13.66 ABa	172.6 ± 14.64 Ba	168.3 ± 13.65 ABb	NT	NT	NT	NT
4	184.6 ± 13.69 ABa	199.01 ± 7.44 ABab	168.3 ± 20.19 ABa	205.7 ± 13.67 Aa	168.6 ± 11.29 ABa	165.4 ± 5.75 Ba	145.7 ± 14.51 ABb	199.1 ± 11.24 Aa	192.9 ± 11.86 Aa	165.4 ± 8.69 CBa	138.0 ± 13.40 Ba
5	217.4 ± 16.12 ABCa	248.0 ± 19.01 ABb	235.4 ± 14.41 ABb	242.3 ± 12.32 Aa	200.0 ± 10.44 CBa	182.6 ± 10.59 Ca	164.6 ± 19.69 Cab	209.1 ± 18.73 ABa	220.0 ± 11.72 ABa	179.3 ± 12.82 Ba	196.0 ± 16.20 ABb
LH (ng/mL)											
1	1.64 ± 0.14 Aa	2.77 ± 0.25 Ba	2.76 ± 0.37 BCa	3.58 ± 0.34 Ba	2.16 ± 0.27 ABa	4.49 ± 0.59 Cac	0.72 ± 0.10 Da	NT	NT	NT	NT
2	4.97 ± 0.71 Abc	3.29 ± 0.33 Ba	6.17 ± 0.44 ACb	6.16 ± 0.89 ACa	7.10 ± 0.55 Cb	2.05 ± 0.37 Db	25.06 ± 1.93 Eb	NT	NT	NT	NT
3	5.44 ± 0.64 Abc	12.62 ± 0.52 Bb	9.46 ± 0.25 Cc	15.66 ± 2.02 BDb	17.50 ± 1.17 Dc	2.87 ± 0.27 Ec	2.85 ± 0.16 Ec	NT	NT	NT	NT
4	6.14 ± 0.42 Ab	5.79 ± 0.52 ACEe	8.59 ± 0.39 Bc	4.16 ± 0.52 Ca	10.17 ± 0.58 Dd	7.24 ± 0.60 Ed	5.08 ± 0.52 Ad	3.91 ± 0.32 Cb	7.96 ± 0.50 Bb	3.17 ± 0.04 Ca	17.08 ± 0.25 Db
5	3.67 ± 0.36 Ac	1.99 ± 0.06 Bd	3.13 ± 0.39 ACa	1.93 ± 0.11 Bc	2.39 ± 0.33 BCa	3.20 ± 0.27 Aac	2.93 ± 0.30 ACc	2.53 ± 0.36 ABc	3.26 ± 0.51 Ac	2.08 ± 0.30 BCb	5.41 ± 0.49 Dc

Capital letters denote statistically significant differences (*p* < 0.05) between the groups in different exposure months, while small letters indicate significant differences in the groups between successive months of exposure

*NT* not tested

After the first month of depuration, body weight in group 4.0 mg Cd/L remained statistically significantly lower in comparison to the control group and group 0.4 mg Cd/L. After 2 months of depuration, we noted a significant body weight increase in fish in this group. The presence of melatonin did not significantly affect fish body weight growth during purification (Table 1).

In the group exposed to the highest cadmium doses, a decrease in locomotive activity and appetite was observed. Melatonin stopped the decrease in locomotive activity in fish from the group exposed to the highest cadmium doses.

### Cd accumulation in the brain

The results of the analysis of cadmium concentration in the brains of Prussian carp are presented in Table 2. Metal accumulation in the brain increased steadily over a 5-month period in four Cd-exposed groups, as confirmed by statistically significant coefficients of correlation (Table 2). A significant increase in Cd concentration was already observed after the first month of the exposure compared to the baseline values in all the experimental groups. The maximum cadmium concentrations were noted at the end of the study (Table 2). After 5 months of exposure, it was observed that in the cadmium-exposed groups (0.4 mg Cd/L + Mel and 4.0 mg Cd/L + Mel), the impact of melatonin resulted in a statistically significant decrease (*p* < 0.05) in Cd accumulation in the brain in comparison to the groups exposed only to the metal (Table 2).

**Table 2:** Comparison of Cd concentration (mg/kg) in the brain tissue, the ovarian tissue, and gonadosomatic index (GSI) of female Prussian carp (mean ± SD) during 5 months of fish exposure to different doses of this metal and 2 month depuration. Spearman’s correlation coefficients (*r*) for the relationship of Cd ovary and brain concentration to cadmium doses during the exposure and depuration periods in different groups. Comparison of Spearman’s correlation coefficients (*r*) for the relationship between GSI value and Cd concentration in gonads. The results of multiple comparisons Kruskal-Wallis test (*p*) for Cd concentration in ovary and GSI value differences

Month	Control	Mel	Blank	0.4 mg Cd/L + Mel	0.4 mg Cd/L	4.0 mg Cd/L + Mel	4.0 mg Cd/L	*r*	Statistic	0.4 mg Cd/L + Mel-dep	0.4 mg Cd/L-dep	4.0 mg Cd/L + Mel-dep	4.0 mg Cd/L-dep	*r*	Statistic
Cd in the brain (mg/kg)															
0	0.28 ± 0.02 Aa	0.64 ± 0.01 Aa	0.45 ± 0.02 Aa	0.65 ± 0.01 Aa	0.36 ± 0.01 Aa	0.65 ± 0.02 Aa	0.52 ± 0.02 Aa	NT	NT	NT	NT	NT	NT	NT	NT
1	0.92 ± 0.05 Abd	0.79 ± 0.04 ABb	0.84 ± 0.03 Ab	0.59 ± 0.08 Bb	0.80 ± 0.04 ABb	0.82 ± 0.04 ABb	1.11 ± 0.11 ABb	0.07	*p* < 0.05	NT	NT	NT	NT	NT	NT
2	0.91 ± 0.01 Ab	0.83 ± 0.08 ABc	0.70 ± 0.19 Bab	1.03 ± 0.06 ABc	1.09 ± 0.06 Ac	1.12 ± 0.10 Cc	1.33 ± 0.08 Cb	0.61***	*p* < 0.05	NT	NT	NT	NT	NT	NT
3	1.11 ± 0.05 Ad	1.20 ± 0.05 ABd	1.33 ± 0.10 BCc	1.22 ± 0.05 ABc	1.49 ± 0.08 CDd	1.54 ± 0.07 CDd	1.82 ± 0.11 Dc	0.65***	*p* < 0.0001	NT	NT	NT	NT	NT	NT
4	1.35 ± 0.07 Ac	1.41 ± 0.08 ABe	1.49 ± 0.06 ABc	1.62 ± 0.08 ABd	1.74 ± 0.08 Bd	1.98 ± 0.05 Ce	2.02 ± 0.10 Cc	0.78***	*p* < 0.0001	1.97 ± 0.19 BCb	1.52 ± 0.08 ABa	1.98 ± 0.12 Cb	2.07 ± 0.10 Ca	0.66***	*p* < 0.0003
5	1.47 ± 0.07 Ac	1.30 ± 0.07 ABde	1.21 ± 0.08 Bc	1.59 ± 0.08 Ad	2.10 ± 0.10 Ce	2.37 ± 0.25 Ce	3.24 ± 0.19 Dd	0.83***	*p* < 0.0001	1.74 ± 0.13 ACab	1.59 ± 0.07 ACa	1.74 ± 0.06 DCb	2.01 ± 0.12 Da	0.71***	p < 0.0001
Cd in the ovary (mg/kg)															
0	0.02 ± 0.01 Aa	0.01 ± 0.01 Aa	0.02 ± 0.01 Aa	0.01 ± 0.01 Aa	0.01 ± 0.00 Aa	0.02 ± 0.01 Aa	0.02 ± 0.01 Aa	NT	NT	NT	NT	NT	NT	NT	NT
1	0.04 ± 0.01 Abcd	0.04 ± 0.01 Abd	0.04 ± 0.01 Ab	0.06 ± 0.01 Bb	0.06 ± 0.00 Bb	0.11 ± 0.02 Cb	0.21 ± 0.03 Db	0.83***	*p* < 0.0001	NT	NT	NT	NT	NT	NT
2	0.03 ± 0.01 Ab	0.03 ± 0.01 Ab	0.03 ± 0.01 Ab	0.17 ± 0.01 Bc	0.20 ± 0.02 Bc	0.63 ± 0.05 Cc	0.91 ± 0.04 Dc	0.94***	*p* < 0.0001	NT	NT	NT	NT	NT	NT
3	0.04 ± 0.01 Abc	0.04 ± 0.00 Abd	0.03 ± 0.00 Ab	0.30 ± 0.01 Bd	0.36 ± 0.09 Bd	1.95 ± 0.24 Cd	4.31 ± 0.80 Dd	0.94***	*p* < 0.0001	NT	NT	NT	NT	NT	NT
4	0.05 ± 0.01 Ac	0.09 ± 0.00 Ac	0.06 ± 0.00 Ac	0.63 ± 0.01 Be	0.57 ± 0.07 Be	5.57 ± 0.44 Ce	8.78 ± 0.84 De	0.94***	*p* < 0.0001	0.46 ± 0.05 Bb	0.46 ± 0.06 Ba	3.93 ± 0.39 Cb	5.749 ± 0.84 Db	0.94***	p < 0.0001
5	0.03 ± 0.01 Ad	0.08 ± 0.02 Bdc	0.07 ± 0.01 Bc	0.71 ± 0.02 Cf	0.76 ± 0.14 Cf	5.87 ± 0.46 De	10.40 ± 1.42 Ef	0.93***	*p* < 0.0001	0.49 ± 0.03 Cb	0.28 ± 0.03 Db	3.64 ± 0.27 Eb	4.39 ± 0.76 Fa	0.94***	p < 0.0001
GSI (%)															
0	10.53 ± 0.73 Aa	9.98 ± 1.29 Aa	10.69 ± 0.59 Aa	9.22 ± 0.48 Aa	10.39 ± 0.74 Aa	10.19 ± 0.57 Aa	10.29 ± 0.59 Aa	NT	NT	NT	NT	NT	NT	NT	NT
1	13.33 ± 1.22 Aa	12.35 ± 1.30 ABa	11.52 ± 0.60 ABa	10.39 ± 0.49 Ba	9.65 ± 1.19 Ba	10.56 ± 1.22 Bab	13.48 ± 0.60 Aa	ns	ns	NT	NT	NT	NT	NT	NT
2	12.16 ± 0.70 Aa	10.87 ± 0.82 ABa	9.56 ± 0.56 Bb	11.19 ± 0.89 ABa	11.42 ± 0.48 Aa	13.48 ± 0.96 Aa	5.57 ± 0.68 Cbc	ns	*p* < 0.0002	NT	NT	NT	NT	NT	NT
3	10.44 ± 0.75 ABa	11.81 ± 0.44 Aa	11.11 ± 0.40 ABab	10.31 ± 0.44 Ba	10.15 ± 0.56 ABa	10.37 ± 0.94 ABb	7.15 ± 0.89 Cb	− 0.49***	*p* < 0.021	NT	NT	NT	- NT	NT	NT
4	6.98 ± 0.51 Ab	4.76 ± 0.50 BCb	5.39 ± 0.63 ABCc	4.06 ± 0.21 CDb	6.16 ± 0.58 ABb	2.98 ± 0.30 Dc	4.83 ± 0.50 Bbc	− 0.49***	*p* < 0.003	5.37 ± 0.78 ABCb	4.26 ± 0.28 BCb	4.03 ± 0.22 Cb	3.33 ± 0.50 Cb	− 0.49***	*p* < 0.0022
5	4.65 ± 0.36 Ac	5.64 ± 0.31 Ab	5.39 ± 0.311 Ac	5.40 ± 0.38 Ac	5.27 ± 0.30 Ab	3.39 ± 0.22 Bc	4.12 ± 0.41 ABc	− 0.39***	*p* < 0.0014	5.16 ± 0.33 Ab	5.29 ± 0.30 Ac	4.21 ± 0.34 ABb	4.49 ± 0.21 BC	− 0.39***	ns

Capital letters denote statistically significant differences (*p* < 0.05) between the groups in different exposure months, while small letters indicate significant differences in the groups between successive months of exposure

*NT* not tested, *ns* not significant

*** *p* < 0.001

The cadmium concentration in fish brains after 3 months of exposure and the subsequent 2-month depuration is shown in Table 2. After the first month of depuration, further growth was observed in the cadmium concentration in the brain of the fish which had been exposed to the highest doses of this metal. However, the accumulation was inhibited after the second month. A significant positive correlation between Cd brain concentration and cadmium dose during the exposure in different groups was noted (Table 2).

### LH concentration

#### Spontaneous LH secretion

After the first month of exposure, a statistically significant (*p* < 0.05) growth in LH concentration was observed in the Mel, blank, 0.4 mg Cd/L + Mel, and 4.0 mg Cd/L + Mel groups compared to the control, 0.4 mg Cd/L, and 4.0 mg Cd/L. In group 4.0 mg Cd/L, a statistically significant decrease in LH concentration was noted compared to the control and the remaining groups. Melatonin in group 4.0 mg Cd/L + Mel significantly inhibited the impact of cadmium on LH secretion (Table 1). After 2 months of exposure, a very high LH level (25 ng/mL) was noted in the group exposed to the highest dose of cadmium (4.0 mg Cd/L). The LH concentration in this group was five times higher than in the control group and was statistically significantly higher in comparison to other groups throughout the remaining experimental period. In group 4.0 mg Cd/L + Mel, the LH concentration in the serum was lower than in the 4.0 mg Cd/L group. In the group of fish exposed to the same Cd dose and to melatonin, the LH concentration in the blood did not exceed 3.0 ng/mL (Table 1).

After 3 months of exposure, the fish entered the spawning period and the level of spontaneous LH secretion increased in the control groups, while in groups 4.0 mg Cd/L + Mel and 4.0 mg Cd/L, a statistically significant lower LH concentration was observed. At the same time, in the group exposed to 0.4 mg Cd/L, a significant increase in the LH concentration was noted in comparison to the remaining groups (Table 1).

After 4 months of exposure in the group exposed to the lowest cadmium dose, the LH level was significantly higher in comparison with the other groups, while in the group exposed to the highest cadmium dose (4.0 mg Cd/L), it remained significantly lower in comparison with groups 4.0 mg Cd/L + Mel, 0.4 mg Cd/L, and blank (Table 1).

After the first month of depuration, a significant (*p* < 0.05) growth in LH concentration was observed in group 4.0 mg Cd/L-dep in comparison with the other groups. In the 0.4 mg Cd/L + Mel-dep and 4.0 mg Cd/L + Mel-dep groups, the LH concentration in the serum was lower than in the 0.4 mg Cd/L-dep and 4.0 mg Cd/L-dep groups.

After the second month of depuration, a decrease in LH concentration was observed in all groups and the fish—also those which had been exposed to cadmium—gradually returned to normal functioning. LH concentration remained statistically significantly higher in the 4.0 mg Cd/L-dep group. Melatonin inhibited the stimulatory effects of Cd on plasma LH levels (Table 1).

#### Hormonal stimulation of LH secretion and ovulation

The highest LH level was found in the group exposed to a cadmium dose of 4.0 mg/L, and it was significantly (*p* < 0.05) higher than in the remaining groups (Table 2S). We also noted an increase in post-injection LH concentration (after 6 and 12 h) in the group exposed to the lowest cadmium dose (0.4 mg Cd/L). Melatonin significantly alleviated Cd-induced elevation of the LH level in groups 0.4 mg Cd/L + Mel and 4.0 mg Cd/L + Mel and after 24 h the LH level in all groups decreased.

Spearman’s correlation coefficients were calculated to determine the relationship between LH secretion and Cd dose during exposure. Positive correlations were noted at 6 h (*r* = 0.38; *p* < 0.01), 12 h (*r* = 0.43; *p* < 0.001) and 24 h (*r* = 0.47; *p* < 0.001) after the stimulating injection (Table 2S).

After 24 h, the ovulation was detected in 67% of females in the group with melatonin implants (Mel). The percentage of ovulating fish was 57, 56, and 17% in the blank, control + sGnRHa, and 0.4 mg Cd/L + Mel groups, respectively. There were no ovulations observed in the groups exposed to 0.4 mg Cd/L, 4.0 mg Cd/L, and 4.0 mg Cd/L + Mel. The percentage of ovulating fish in the blank, control + sGnRH, Mel, and 0.4 mg Cd/L + Mel groups was statistically significantly higher in comparison to the 0.4 mg Cd/L, 4.0 mg Cd/L, and 4.0 mg Cd/L + Mel groups (Table 2S).

### Cd accumulation in ovaries

The results of the cadmium concentration analysis in the ovaries of Prussian carp are shown in Table 2. A statistically significant (*p* < 0.05) increase in the Cd concentration in the ovaries was already noted in the first month of exposure to cadmium in comparison with the 0 (baseline) value and continued to increase until the end of the experiment, depending on the exposure dose, which was confirmed by a statistically significant positive correlation coefficient (Table 2). The cadmium concentration in the ovaries of the exposed fish increased every month, reaching the maximum level after the fifth month of exposure. In the group of females which were exposed to the highest cadmium doses (4.0 mg Cd/L), a statistically significant increase in cadmium concentration was observed compared to the control and the remaining groups in every sample (month). Melatonin in group 4.0 mg Cd/L + Mel had a statistically significant inhibitory effect on Cd accumulation in fish gonads in every month (sample) of the experiment (Table 2).

The results of Cd concentration analysis after 3 months of exposure and 2 months of depuration are presented in Table 2. After the first month of depuration, a further statistically significant (*p* < 0.05) increase in Cd concentration was observed in the gonads of the fish which had been exposed to the highest dose of this metal. During the depuration phase, Spearman’s correlation coefficients were significant positive, confirming the relationship of Cd ovary level with its previous exposure level (Table 2). In the depuration period, melatonin caused a statistically significant Cd concentration decrease in the gonads of fish exposed to the highest cadmium dose after both the first and second months of depuration (Table 2).

### GSI

After the second and third months of exposure, a significant (*p* < 0.05) decrease in GSI was noted in the group exposed to the highest cadmium dose (4.0 mg Cd/L) compared to the other groups. In the 4.0 mg Cd/L + Mel group, melatonin significantly inhibited the impact of cadmium on GSI (Table 2). In the fourth and fifth months, a statistically significant decrease in GSI level was noted in all groups compared to the previous sample collections. This might have been caused by spawning, which took place in the third month of exposure (Table 2).

During depuration, a statistically significant decrease in GSI was noted in all groups (Table 2). A negative correlation between GSI and Cd concentration in the gonads was observed after a 3-month period (*r* = − 0.49; *p <* 0.001), a 4-month period (*r* = − 0.49; *p <* 0.001), and after a 5-month period of exposure (*r* = − 0.39; p < 0.001) (Table 2).

## Discussion

The study shows that most likely melatonin has a preventive effect on the toxicity of cadmium at each level of the brain-ovary axis.

### Accumulation

Changes in the physiological processes controlled by the hypothalamus and the pituitary gland may be caused by the accumulation of cadmium in both of these structures (Lafuente et al. 2001). During the course of the present study, we observed a significant increase in cadmium concentration in the brains of fish in the group exposed to the highest dose of this metal after 5 months of exposure (Table 2). A positive correlation was also found between the cadmium concentration in the brain and the duration of exposure. Such an increase was also noted by Allen (1995) in the study on *Oreochromis aureus* after a long-term exposure to cadmium in water. Similar results were obtained by other authors investigating various fish species (Ladhar et al. 2014). The brain is peculiarly sensitive to Cd toxicity. The accumulation of this metal in the brain leads to histopathological changes, i.e., tissue disorganization, mitochondria swelling with a loss of cristae, vacuolization, and dystrophy (large autophagic vacuoles) (Favorito et al. 2011; Al-sawafi et al. 2017). Moreover, cadmium causes hyperemia, along with cell and axon necrosis in the brain (Kaoud and Mekawy 2011). Kumari and Dutt (1991) demonstrated overgrowth and vacuolization of the pituitary gland cells in *Puntius sarana*. The present research demonstrated a preventive effect of melatonin on the brain. Therefore, it probably protects the brain from the neurotoxic effects of cadmium. The mechanisms underlying the decrease in cadmium concentration caused by melatonin are unknown. One possibility is that melatonin is able to create stable complexes with cadmium (Limson et al., 1998), which allows the metal to be captured in the gills or intestines, where melatonin is stored (Kulczykowska et al. 2006). Chwełatiuk et al. (2006) proved in their research that administering cadmium with melatonin decreased the concentration of this metal in the kidney, liver, and intestines of mice. Additionally, melatonin is highly lipid-soluble and is transported freely across the blood-brain barrier and cellular membranes. In consequence, it is easier for melatonin to remove cadmium directly from tissues. Moreover, being an intracellular antioxidant, it can protect cells directly from the damaging effect of free radicals (Eybl et al. 2006).

The presence of cadmium in the brain and its neurotoxic properties disrupt the synthesis and secretion of neurotransmitters in the hypothalamus and pituitary gland of the rat (Pillai et al. 2003; Lafuente et al. 2005). Changes in the release of LH gonadotropin from the pituitary gland under the influence of cadmium were also observed in our study. After 2 months of exposure, the LH concentration in fish blood was significantly higher in the group exposed to the highest dose of cadmium (Table 1). Similar results were obtained by Szczerbik et al. (2006) and Mikołajczyk et al. (1990). These observations indicate that an increase in LH secretion was one of the most important effects of this metal on fish reproduction. One of the possible reasons for that could be the direct toxic effect of cadmium on the hypothalamus and the pituitary gland. Numerous studies conducted on mammals confirm that cadmium affects the activity of the hypothalamus and the pituitary gland. The inhibiting effect of cadmium on releasing biogenic amines from the hypothalamus of the rat has been demonstrated by many authors (Pillai et al. 2003; Lafuente et al. 2005; Romero et al. 2011). Cadmium indirectly stimulates the release of LH from the pituitary gland by inhibiting the release of dopamine, functioning as a gonadotropin release-inhibiting factor in fish. The direct inhibitive effect of cadmium on the release of hormones from the pituitary gland has been observed in numerous studies on mammals (Lafuente and Esquifino 1999; Lafuente et al. 2004). By causing an increase in reactive oxygen species in the pituitary gland, cadmium leads to necrosis and apoptosis of cells (Poliandri et al. 2006). The present study is the first to observe a preventive effect of melatonin against the neurotoxic influence of cadmium in fish at the level of the brain. Melatonin most likely inhibited the negative effect of cadmium on the release of LH. So far, the mechanism by which melatonin provides protection against the neurotoxic influence of cadmium on the release of gonadotropins has not been explained. It is known that melatonin can directly affect the hypothalamus and the pituitary gland of fish, since a high number of melatonin receptors (MT1 and MT2) have been found in these organs (Falcón et al. 2007; Sauzet et al. 2008). A study by Carretero et al. (2009) indicates that melatonin reduces mitochondrial disorders in the brain and reduces the level of free radicals by stimulating antioxidant enzyme activity (Rodriguez et al. 2004; Tomas-Zapico and Coto-Montes 2005). Furthermore, melatonin reduces cadmium-induced nitric oxide synthase expression and lipid peroxidation in the hypothalamus and pituitary gland in rats (Poliandri et al. 2006). In addition, melatonin has anti-apoptotic properties (Tuñón et al. 2011). However, further studies are needed to clarify the melatonin’s antioxidant mechanism of action.

The present study additionally assessed the impact of cadmium and melatonin on hormonal stimulation of spawning in Prussian carp females (using salmon GnRH analogue and pimozide). In groups of fish exposed to a low or high cadmium concentration in water, a significant increase in LH concentration in the blood plasma of females was detected after the stimulation (Table 2S). No ovulation was observed in these groups. These results indicate that cadmium has a negative impact on the ovulation process in fish. The effects observed also confirm the results obtained on the same species by Szczerbik et al. (2006), who noted that cadmium added to the feed has a stimulating effect on LH secretion under the influence of hormonal stimulation and at the same time inhibits ovulation. In our research, melatonin most likely inhibited the negative impact of cadmium on LH release in groups exposed to both low and high concentrations of this metal. Although melatonin prevented excessive LH secretion induced by the presence of cadmium in the brain, the effect was not reflected in ovulation. This suggests that melatonin may act more strongly at the level of the pituitary gland than at the level of the ovaries.

Cadmium not only influences secretion of hormones in the hypothalamus and the pituitary gland, but also affects the gonads by disrupting the synthesis and release of steroid hormones (Alonso-González et al. 2007; Ji et al. 2010). We observed a significant increase in the cadmium concentration in the gonads of fish exposed to the highest dose of this metal. It was shown that more cadmium accumulated in the ovaries than in the brain (Table 2). Similar results were obtained by other authors (Allen 1995; Kim et al. 2009). Since cadmium accumulates in the gonads in such large quantities, it may directly disturb their functioning and structure. El-Ebiary et al. (2013) concluded that cadmium directly affects gonad structure through vacuolization disorders (abnormally shaped ova with a great number of vacuoles and a detached follicular wall). Changes in the ovary of *Heteropneustes fossilis* were also observed by Sharma et al. (2011). These changes included degeneration in the ovarian follicles, as well as a change in the nucleus and in the appearance of atretic follicles. One of the possible reasons for this state may be vitellogenesis disruption. This hypothesis is confirmed by studies by other authors. Pundir and Saxena (1992) demonstrated that cadmium most probably acts at the stage of vitellogenesis and at the final stages of oocyte maturation by disrupting synthesis of reproductive hormones responsible for the development and ovulation of egg cells. In an in vitro study, Hwang et al. (2000) showed that cadmium inhibits the synthesis of vitellogenin in the hepatocytes of the rainbow trout. Pereira et al. (1993) noted that cadmium caused decreased vitellogenin levels in the blood of the flounder. In the tests carried out on the common carp during vitellogenesis, Das and Mukherjee (2013) observed that cadmium gradually attenuated spontaneous secretion of 17β-oestradiol. Szczerbik et al. (2006) suggested that in the Prussian carp, cadmium acts mainly at the level of the ovary, not the pituitary gland. Additional exposure to cadmium impairs gametogenesis. Oogenesis was delayed in the brown trout and inhibited in the rainbow trout after exposure to this metal (Brown et al. 1994). This means that cadmium affects the quantity and quality of gametes (Annabi et al. 2012). Our study is the first to observe a preventive effect of melatonin on the ovaries of fish exposed to cadmium in water. Numerous studies have shown that melatonin regulates steroidogenesis, folliculogenesis, and the maturation of oocytes in mammalian ovaries (Tanavde and Maitra 2003). Studies conducted on fish in recent years have demonstrated the presence of melatonin receptors in fish ovaries (Chattoraj et al. 2009; Chai et al. 2013). We also know that melatonin plays an important role in fish reproduction by modulating the function of steroid hormones in the final stage of oocyte maturation (Maitra et al. 2013). In carp, melatonin stimulates oocyte maturation, causing germinal vesicle breakdown GVBD (Popek et al. 1996). Melatonin also plays an important role in regulating vitellogenesis in fish (Popek et al. 1997; Mazurais et al. 2000). In addition, experiments on vertebrates have shown that melatonin and its metabolites have a potentially beneficial impact on pathophysiological changes in the ovaries (Pai and Majumdar 2014). Removing the pineal gland in rats led to the development of follicular cysts, increased ovarian follicle atresia, ovarian stromal hypertrophy, and endometrial hyperplasia (Singh 2005). However, the mechanism of melatonin protection against the toxic effect of cadmium on fish gonads is not known. It is probable that melatonin, being a strong antioxidant, directly affects the ovaries by penetrating into egg cells and protecting them from the harmful effects of reactive oxygen species, which is similar in the case of the brain (Pai and Majumdar 2014). Melatonin’s ability to capture cadmium may be another protection mechanism (Limson et al. 1998; Chwełatiuk et al. 2006).

The toxic effect of cadmium on the gonads is evidenced by its influence on the GSI level. The GSI is a tool for measuring sexual maturity of animals in correlation to ovary development. We found that Cd reduced the GSI in the group exposed to the highest dose of cadmium (Table 2). The drop in GSI caused by cadmium was also noticed by other authors (Pereira et al. 1993; Szczerbik et al. 2006). Cadmium added to the feed of zebrafish and sheepshead minnows reduced the percentage of live eggs and impeded their production (Karels et al. 2003).

In our experiment, melatonin inhibited the negative effect of cadmium on GSI in the group receiving the highest dose of cadmium. One of the possible reasons for that is the direct toxic effect of cadmium on the gonads. In *Danio rerio*, melatonin caused an increase in GSI associated with higher egg production and with elevated protein and gene levels of vitellogenin and estradiol receptors in the liver (Carnevali et al. 2011). Another possible cause may be the indirect influence of melatonin on the synthesis and release of vitellogenin from the liver, where the hormone is stored (Messner et al. 2001).

### Mortality, body weight, and behavior

Changes in hypothalamic concentration of neurotransmitters caused by cadmium may have adverse effects on various life processes as the hypothalamus participates in controlling and regulating body temperature, sexual and locomotor activity, hunger, and satiety (Romero et al. 2011). The present study revealed a decrease in fish body weight along with exposure time in fish exposed to cadmium. In addition, we observed increased mortality in groups exposed to cadmium (Table 1). The inhibitory effect of cadmium on fish growth may be caused by disturbances in food intake and assimilation. The negative impact of cadmium on growth, body weight, and mortality has also been acknowledged by other authors (Szczerbik et al. 2006; Annabi et al. 2012; El-Serafy et al. 2013). Melatonin did not affect body weight, but it clearly reduced fish mortality, since there were no cases of death in the groups of fish exposed to cadmium with implants that continuously released melatonin. It may be assumed that one of the reasons for the reduction in the mortality rate was melatonin’s immunostimulating effect (Singh et al. 2015).

Changes in fish locomotor activity may increase or decrease chances of survival in a changing natural environment (Little and Brewer 2001). The present study shows the inhibition of locomotor activity in fish exposed to the highest concentration of cadmium in water. Our results are in agreement with the results reported by Yilmaz et al. (2004), who also observed behavioral changes in fish exposed to cadmium, such as swimming in imbalanced manner, capsizing, attaching to the surface, difficulty in breathing and gathering around the ventilation filter, slowness in motion, and sinking down to bottom. Also, Szczerbik et al. (2006) showed a reduction in locomotor activity in the Prussian carp fed with cadmium-contaminated feed. In this study, melatonin counteracted the inhibitive effect of cadmium on locomotor activity of fish, which was reflected in greater freedom of breathing and swimming, and increased mobility. In the study by Singh et al. (2015), melatonin reduced aggression in *Channa punctatus* induced by exposure to cadmium. Melatonin, which is considered to be an anti-stress hormone, mitigates the effects of stress and increases resistance in fish (Singh et al. 2015; Jung et al. 2016).

### Depuration

Elimination of cadmium from tissues after 3 months of exposure depends on the duration of elimination time and tissue type. During 2 months of depuration, there was a significant increase in the cadmium concentration in the brain as well as in the gonads in the groups of fish exposed to this metal. Slow elimination of Cd was observed in both the brain and the gonads in the second month of depuration. In the melatonin-implant group, there was also an increase in cadmium concentration in the ovaries during the depuration phase, but this increase was significantly lower than in the group without melatonin (Table 2). Kim et al. (2004) observed different levels of cadmium elimination from tissues in *Paralichthys olivaceus* after exposure to this metal. Slow elimination of cadmium from the muscles and the liver was noted by Kargin and Çoğun (1999). The increase in the cadmium concentration in the gonads during depuration was probably the result of an increase in the concentration of cadmium in blood due to the release of the metal from other tissues. After the exposure to cadmium has ended, the metal accumulated in various tissues of the organism is gradually released into the bloodstream. Afterwards, it may be excreted from the organism or accumulated once again in more sensitive tissues (Łuszczek-Trojnar et al. 2013). Interestingly, an increase in LH concentration was also found in the group exposed to the highest cadmium concentration for 3 months. This increase probably stemmed from an elevated level of cadmium in the blood as a result of its elimination from tissues that had accumulated a large amount of this metal. It is likely that cadmium had a neurotoxic effect at the level of the brain by disturbing synthesis and release of neurotransmitters. In contrast, melatonin inhibited the neurotoxic effect of cadmium in the group exposed to the highest dose of the metal by preventing excessive LH release from the pituitary gland (Table 1).

## Conclusion

Our study indicates that one of the most significant effects of cadmium is raising the LH level in the blood plasma in the group of females exposed to the highest dose of this element, with simultaneous visible delay in gonad maturation, observed as a decrease in the GSI value, and increase in the cadmium concentration in both the brain and the ovaries. In all cases, the negative effects of cadmium were most likely inhibited by melatonin. The effects of melatonin point to a possible role of this indoleamine as a preventive agent for environmental Cd contamination. Melatonin provides protection against the toxic effect of cadmium on crucial processes, such as maturation and reproduction in both farmed and wild fish, and thus may increase the size of a population and improve the survival of a species.

## Electronic supplementary material

ESM 1 (DOC 64 kb)
