# Supplementary material for: Protective effect of melatonin on cadmium-induced changes in some maturation and reproductive parameters of female Prussian carp (Carassius gibelio B.)
Source: Environ Sci Pollut Res Int. 2018 Jan 26;25(10):9915–27. doi: 10.1007/s11356-018-1308-8 (PMC5891563; doi:10.1007/s11356-018-1308-8)
Supplement: Supplementary file 1 — (DOC 64 kb) [file 11356_2018_1308_MOESM1_ESM.doc]

Supplementary material

Protective effect of melatonin on cadmium-induced changes in some maturation and reproductive parameters of female Prussian carp (*Carassius gibelio* B.)

Ewa Drąg-Kozak*^1^, Magdalena Socha^1^, Grzegorz Gosiewski^1^, Ewa Łuszczek-Trojnar^1^, Jarosław Chyb^1^, Włodzimierz Popek^1^

1. Department of Ichthyobiology and Fisheries, University of Agriculture in Krakow, ul. Spiczakowa 6, 30-199 Kraków-Mydlniki, Poland

*Corresponding autor.

Department of Ichthyobiology and Fisheries, University of Agriculture in Krakow, ul. Spiczakowa 6, 30-199 Kraków-Mydlniki, Poland

E-mail address: [ewa.drag-kozak@urk.edu.pl](mailto:ewa.drag-kozak@urk.edu.pl) (E. Drąg-Kozak)

Table 1S. The configuration of treatment groups and cadmium doses in water during the 3 months exposure period and the following 2 months exposure or depuration period.

| Group | control | Mel | blank | 0.4 mgCd/L+Mel | | 0.4 mgCd/L | | 4.0 mgCd/L+Mel | | 0.4 mgCd/L | |
| --- | --- | --- | --- | --- | --- | --- | --- | --- | --- | --- | --- |
| Cd dose in water (mg/L) during 1- 3 months period | - | - | - | 0.4 | | 0.4 | | 4.0 | | 4.0 | |
| Number of fish at the beginning [n] (per tank) | 49 | 49 | 49 | 49 | | 49 | | 49 | | 49 | |
| Group  During 4-5 months of experiment | control | Mel | blank | 0.4 mgCd/L+Mel | 0.4 mgCd/L+Mel-dep | 0.4 mgCd/L | 0.4 mgCd/L - dep | 4.0 mgCd/L+Mel | 4.0 mgCd/L+Mel - dep | 4.0 mgCd/L | 4.0 mgCd/L - dep |
| Cd dose in water (mg/L) during the 4-5 months period | - | - | - | 0.4 | - | 0.4 | - | 4.0 | - | 4.0 | - |
| Number of fish at the beginning of the 4 months exposure | 14/14 | 14/14 | 14/14 | 14 | 14 | 14 | 14 | 14 | 14 | 14 | 14 |

Table 2S. The results of statistical analysis for LH plasma concentration at 0, 6, 12 and 24 hours after the stimulating injection of fish exposed to different doses of Cd and/or melatonin (Mann-Whitney test between each groups and multiple comparisons Kruskal-Wallis test between all groups). Comparison of Spearman’s correlation coefficients (*r*) for the relationship between spontaneous or stimulated LH blood plasma level and Cd water concentration during exposure. Comparison of percentages of ovulation in particular groups after 24 h post injection.

|  | LH concentration (ng/mL) after 3 months Cd exposure | | | | Ovulation |
| --- | --- | --- | --- | --- | --- |
| Hours (h) | 0 | 6 | 12 | 24 | After 24h |
| Control | 5.4±0.53  Aa | 8.25±0.35  Ab | 8.60±0.49  Ab | 3.10±0.32  Ac | no |
| Control+S | 8.88±0.51  Ba | 37.76±0.32  Bb | 69.71±0.67 | 34.06±0.3 | 56%# |
| Mel | 12.62±0.52  Ca | 54.95±3.27 | 130.79±11.58 | 45.69±3.46 | 67%# |
| blank | 9.46±0.41  Ba | 46.09±0.44  Db | 78.31±5.79  Bc | 47.16±3.94  Db | 57%# |
| 0.4 mgCd/L+Mel | 15.66±0.86  Da | 26.61±1.67  Eb | 115.94±6.80  Cc | 70.86±5.17  Cd | 17%# |
| 0.4 mgCd/L | 17.50±1.17  Da | 144.12±5.51  Fb | 147.07±11.72  Cb | 31.82±2.81  Dc | no |
| 4.0 mgCd/L+Mel | 3.36±0.26  Ea | 43.11±4.87  Gb | 63.12±12.30  Bc | 57.09±1.84 | no |
| 4.0 mgCd/L | 2.85±0.16  Ea | 157.52±0.40  Fb | 205.35±5.76  Dc | 48.93±5.46  Bd | no |
| Kruskal-Wallis test | p<0.0001 | p<0.0001 | p<0.0001 | p<0.0001 | NT |
| *r* | -0.33* | 0.38** | 0.43*** | 0.47*** | Nt |

*p<0.05; **p<0.01;***p<0.001

*Capital letters* denote significant differences (p<0.05) between the groups, *small letters* indicate significant differences in the groups between successive hours after stimulation. # p<0.05 significant differences between groups. NT- not tested.
